# Maintenance therapy for newly diagnosed epithelial ovarian cancer– a review

**DOI:** 10.1186/s13048-022-01020-1

**Published:** 2022-07-28

**Authors:** Shona Nag, Shyam Aggarwal, Amit Rauthan, Narayanankutty Warrier

**Affiliations:** 1 Sahyadri Speciality Hospitals, Pune, Maharashtra India; 2 Sir Gangaram Hospital, New Delhi, India; 3 Manipal Hospital, Karnataka Bangalore, India; 4 MVR Cancer Centre and Research Institute, Calicut, Kerala India

**Keywords:** Anti-angiogenic agents, Epithelial ovarian cancer, Maintenance therapy, Molecular-targeted therapy, PARP inhibitors

## Abstract

Epithelial ovarian cancer (EOC) is the most lethal gynaecological cancer among women worldwide, with the 5-year survival rate ranging between 30 and 40%. Due to the asymptomatic nature of the condition, it is more likely to be diagnosed at an advanced stage, requiring an aggressive therapeutic approach. Cytoreductive surgery (CRS) along with systemic chemotherapy with paclitaxel and carboplatin has been the mainstay of the treatment in the frontline management of EOC. In recent years, neo-adjuvant chemotherapy, followed by interval CRS has become an important strategy for the management of advanced EOC. Due to the high rate of recurrence, the oncology community has begun to shift its focus to molecular-targeted agents and maintenance therapy in the frontline settings. The rationale for maintenance therapy is to delay the progression or relapse of the disease, as long as possible after first-line treatment, irrespective of the amount of residual disease. Tumours with homologous recombination deficiency (HRD) including BReast CAncer gene (*BRCA)* mutations are found to be sensitive to polyadenosine diphosphate-ribose polymerase (PARP) inhibitors and understanding of HRD status has become important in the frontline setting. PARP inhibitors are reported to provide a significant improvement in progression-free survival and have an acceptable safety profile. PARP inhibitors have also been found to act regardless of *BRCA* status. Recently, PARP inhibitors as maintenance therapy in the frontline settings showed encouraging results in EOC; however, the results from further trials and survival data from ongoing trials are awaited for understanding the role of this pathway in treatment of EOC. This review discusses an overview of maintenance strategies in newly diagnosed EOC along with considerations for maintenance therapy in EOC with a focus on PARP inhibitors.

## Introduction

Ovarian cancer (OC) is a lethal gynaecological cancer, with 313,959 new cases and 207,252 deaths, worldwide in 2020 [1]. Among Indian women, OC ranks third after cervical and uterine cancer accounting for approximately 45,701 new cases and 32,077 deaths [1, 2].

Epithelial ovarian cancer (EOC) accounts for over 90% of the OC cases [3]. EOC develops in two different oncogenic pathways. The vast majority follow the type II pathway, present with p53 and BReast CAncer gene (*BRCA*) mutations, and are high grade serous tumors. Whereas, low-grade serous tumors are characterized by *BRAF*, *KRAS*, *PTEN*, *PIK3CA*, *ARID1A*, *CTNNB1*, and *PPP2R1A* mutations and progress according to the type I pathway [4]. Due to non-specific symptoms, the disease is usually diagnosed at an advanced stage resulting in a 5-year survival rate ranging between 30 and 40% across the globe, even with optimal care [5].

Cytoreductive surgery (CRS) along with systemic chemotherapy with paclitaxel and carboplatin has been the mainstay of the treatment in the frontline management of EOC for the last 20 years. In recent years, neo-adjuvant chemotherapy, followed by an interval CRS has become an important strategy for the management of advanced OC [6]. In advanced EOC, more than 70% of the patients eventually relapse within 3 years of first-line treatment [7, 8]. With disease progression, other complications such as ascites, bowel obstruction and pleural effusion arise affecting the quality of life. Thus, delaying recurrence or progression of disease and improving survival following first-line treatment is still a significant unmet need in patients with EOC.

At the time of diagnosis, approximately 50% of EOCs exhibit deficiency to repair deoxyribonucleic acid breaks due to alterations (epigenetic and genetic) in homologous recombination repair (HRR) pathway genes [9]. The most prominent one is *BRCA* mutations in tumour suppressor gene, which accounts for almost 18% of EOC cases [10]. In EOC, germline *BRCA (gBRCA*) mutations are identified in 13 to 15% of the cases and somatic *BRCA* mutations are found in 5 to 10% of the cases [11, 12]. The incidence of *gBRCA* mutation varies widely based on the ethnicity (8 to 17% in Caucasians compared with 15 to 30% in Asians) [13–18]. Mutations that interfere with normal function of *BRCA* are reported to modulate outcomes of treatment with platinum/molecular-targeted drugs [19, 20].

Molecular-targeted drugs─ antiangiogenic agents have demonstrated encouraging results in patients with newly diagnosed advanced OC following first-line treatment [21]. Based on the results of these studies, National Comprehensive Cancer Network^®^(NCCN^®^) recommends bevacizumab for targeted therapy with platinum-based chemotherapy and maintenance monotherapy as options in the frontline setting for certain patients with advanced EOC [22]. Post-chemotherapy, maintenance treatment with polyadenosine diphosphate-ribose polymerase (PARP) inhibitors has shown promising results with recurrent disease [23–27]. PARP inhibitors are also recommended as frontline maintenance treatment options for certain patients with EOC [22, 23, 28].

This review explores maintenance therapy as a strategic approach for extended disease control with the intention of prolonging survival in management of newly diagnosed EOC in frontline settings.

## Overview of maintenance strategies in epithelial ovarian cancer

Although first-line platinum-based chemotherapy regimen has remained a mainstay in the treatment of EOC, the progression-free survival (PFS) remains poor (< 2 years) necessitating second-line therapies [7, 29–31]. The ICON-3 study conducted on patients with histologically confirmed invasive EOC has reported a high relapse rate of above 60% with paclitaxel plus carboplatin regimen [31]. The median PFS period reported in this study was of 17.3 months and median overall survival (OS) of 36.1 months with carboplatin plus paclitaxel regimen [31]. In this context, the maintenance therapy is being studied to delay the progression or relapse of the disease, as long as possible after first-line surgical treatment, irrespective of the amount of residual disease.

### Chemotherapeutic agents

Clinical studies (GOG-178 [32], MITO-1 [33], AGO-GINECO [34] and After-6 [35]) examined the efficacy of maintenance treatment with chemotherapeutic agents, 12-cycles of paclitaxel, topotecan, sequential addition of topotecan to carboplatin–paclitaxel, 6-cycles of paclitaxel, respectively after the first-line chemotherapy in improving the prognosis in patients with OC. Studies have revealed a PFS gain of approximately 6 to 8 months when compared with patients who did not receive maintenance therapy, but reported more toxicity and failed to demonstrate survival benefit.

### Antiangiogenic drugs

Vascular endothelial growth factor (VEGF) promotes angiogenesis and vascular permeability leading to malignant effusion and disease progression. Patients with high circulating serum levels of VEGF are at an increased risk of disease recurrence and death [36]. The United States (US) Food and Drug Administration (FDA) initially approved bevacizumab, an antiangiogenic drug, in combination with chemotherapy for platinum-resistant recurrent EOC in patients who received no more than two prior chemotherapy regimens based on the results from AURELIA trial [37]. In platinum-sensitive recurrent EOC, bevacizumab was approved in combination with either carboplatin and paclitaxel or carboplatin and gemcitabine, followed by bevacizumab as a single agent, based on the findings from two randomised phase III trials, GOG-0213 [38] and OCEANS [39].

In 2018, the US FDA approved bevacizumab in combination with chemotherapy (carboplatin and paclitaxel), followed by a single agent bevacizumab as maintenance for patients with stage III or IV EOC, after initial surgical resection based on GOG-0218 [40] trial results. GOG-0218 reported an improvement in PFS in patients who received bevacizumab plus chemotherapy followed by bevacizumab maintenance therapy compared with patients who received platinum-based chemotherapy alone (14.1 months vs. 10.3 months, respectively) and no difference was observed in the overall population, in the final protocol-specified analyses [40]. However, in post-hoc subgroup analyses, a significant OS benefit was observed with bevacizumab-concurrent plus maintenance compared with chemotherapy alone in patients with stage IV disease (42.8 months vs. 32.6 months, hazard ratio [HR]: 0.75; 95% confidence interval [CI], 0.59 to 0.95) [41]. ICON-7 study also showed a modest PFS benefit with bevacizumab in patients with less advanced disease (17.3 months vs. 19.8 months, *p* < 0.004) [21]. However, in high-risk patients (stage III with > 1 cm residual disease or stage IV) a significant improvement in PFS (18.1 months vs. 14.5 months) was observed with corresponding improvement in OS, in an exploratory analyses (39.7 months vs. 30.2 months) [42]. The single-arm ROSiA study reported improved PFS (25.5 months, 95% CI, 23.7 to 27.6 months) with extended use of bevacizumab (continued until progression or for up to 24 months) in combination with paclitaxel after debulking surgery [43].

In BOOST trial (phase III trial) involving patients with stage IIB–IV disease, who underwent primary CRS followed by six cycles of chemotherapy (paclitaxel+carboplatin) and bevacizumab, longer treatment with bevacizumab with carboplatin and paclitaxel for up to 30 months have neither showed PFS nor OS benefit [44].

Several other antiangiogenic drugs such as pazopanib, sorafenib, nintedanib and trebananib have been investigated for the management of EOC; however, none of them have been granted approval due to safety concerns (Table 1) [21, 40–43, 45–49]. Bevacizumab remains an only antiangiogenic drug in market for the treatment of EOC in both frontline as well as in recurrent settings.

**Table 1:** Clinical trials of antiangiogenic agents in maintenance therapy in EOC (frontline settings)

Study	Trial design and no of patients (n)	Patient population	Study arms	Median PFS/OS, months (HR, 95% CI)	Conclusions
GOG-0218 [40, 41]	Double-blind, placebo-controlled (*n* = 1873)	Newly diagnosed stage III or IV EOC with gross residual disease after maximal debulking effort	Arm 1: Carboplatin AUC 6, paclitaxel 175 mg*/*m^2^ q3 weeks for six cycles + placebo q3 weeks cycles 2–22 Arm 2: Carboplatin AUC 6, paclitaxel 175 mg*/*m^2^ q3 weeks for six cycles + Bevacizumab 15 mg*/*kg q3 weeks cycles 2–6 and placebo q3 weeks cycles 7–22 Arm 3: Carboplatin AUC 6, paclitaxel 175 mg*/*m^2^ q3 weeks for six cycles + Bevacizumab 15 mg*/*kg q3 weeks cycles 2–22	**mPFS:** Arm 1: 10.3 Arm 2: 11.2 (HR: 0.91, 0.80–1.04, *p* = 0.16) Arm 3: 14.1 (HR: 0.72, 0.63–0.82, *p* < 0.001) **mOS:** Arm 1: 41.1 Arm 2: 40.8 (HR: 1.06, 0.94–1.20, *p* = 0.34) Arm 3: 43.4 (HR: 0.96, 0.85–1.09, *p* = 0.53) Exploratory subset analyses: Stage III disease: Arm1: 44.2 Arm 2: 42.9 (HR: 1.08, 0.93–1.25) Arm 3: 44.3 (HR: 1.05, 0.92–1.20, *p* = 0.49) Stage IV disease: Arm1: 32.6 Arm2: 34.5 (HR: 0.99, 0.78–1.26) Arm 3: 42.8 (HR: 0.75, 0.59–0.95)	The use of bevacizumab during and up to 10 months after carboplatin and paclitaxel chemotherapy prolongs the PFS by about 4 months. In ITT population, no survival differences were found between patients receiving bevacizumab compared to chemotherapy alone. Patients with FIGO stage IV disease may derive a survival advantage from bevacizumab when administered with and following frontline chemotherapy.
ICON7 [21, 42]	Open-label study (*n* = 1528)	Newly diagnosed stage I–IIA grade 3 EOC, any stage with clear cell histology and stage III or IV EOC all after maximal debulking effort	Arm 1: Carboplatin AUC 5 or 6, paclitaxel 175 mg*/*m^2^ q3 weeks for six cycles + placebo q3 weeks cycles 1 or 2 through cycle 18 Arm 2: Carboplatin AUC 5 or 6, paclitaxel 175 mg*/*m^2^ q3 weeks for six cycles + Bevacizumab 7.5 mg*/*kg q3 weeks cycles 1 or 2 through cycle 18	**mPFS:** Total cohort Arm 1: 17.5 Arm 2: 19.9 (HR: 0.93, 0. 83–1.05, *p* < 0.001) High-risk progression^a^ Arm 1: 10.5 Arm 2: 16.0 (HR: 0.73, 0.60–0.93, *p* < 0.001) **mOS:** Total cohort Arm 1: 58.6 Arm 2: 58.0 (HR: 0.99, 0.85–1.14, *p* = 0.02) High-risk progression^a^ Arm 1: 30.2 Arm 2: 39.7 (HR: 0.78, 0.63–0.97, *p* = 0.01)	Bevacizumab improved PFS in women with OC. The benefits with respect to both PFS and OS were greater among those at high-risk for disease progression.
ROSiA [43]	Single-arm study (*n* = 1021)	Stage IIB to IV or grade 3 stage I to IIA OC without clinical signs or symptoms of gastrointestinal obstruction or history of abdominal fistula, gastrointestinal perforation or intra-abdominal abscess within the preceding 6 months	Bevacizumab (15/7.5 mg/kg) q3w, 4–8 cycles of paclitaxel (investigator’s choice of 175 mg/m^2^ q3w or 80 mg/m^2^ weekly) plus carboplatin AUC 5– 6 q3w	**mPFS:** 25.5 (23.7–27.6) In high-risk disease: 18.3 (16.8–20.6) In non-high-risk disease: 32 (30.9–40.2) **mOS:** The 2-year OS rate was 85% (83–87%)	The median PFS of 25.5 months is the longest reported to date. OS results are immature with events in only 23% of patients. Extended bevacizumab-containing therapy is both tolerable and feasible.
Herzog et al. study [45]	Randomised, double-blind, placebo-controlled, phase IIB study	Women with advanced stage EOC or primary peritoneal cancer who achieved clinical complete response after tumour debulking and one regimen of standardized platinum/taxane-containing therapy	Arm 1: Sorafenib 400 mg or BID) Arm 2: Placebo	**mPFS:** Arm 1:12.7 (HR: 1.09, 0.72 –1.63) Arm 2: 15.7	No significant difference between sorafenib and placebo arms for PFS.
AGO-OVAR16 [46, 47]	Randomised, double-blind, placebo-controlled, phase III trial (*n* = 940)	Histologically confirmed OC stages II-IV who have not progressed after first-line chemotherapy	Arm 1: Pazopanib (800 mg once daily) Arm 2: Placebo	**mPFS:** Arm 1:17.9 (HR: 0.77, 0.64 –0.91, *p* = 0.0021) Arm 2: 12.3 **mOS:** Arm 1: 59.1 (HR: 0.96, 0.805 –1.145, *p* = 0.6431) Arm 2: 64.0	Pazopanib maintenance therapy prolonged PFS; however, no difference in OS was observed between pazopanib and placebo. At the time of the final OS analyses, 494 (89.7% of the planned 551) events had occurred.
AGO-OVAR12 [48]	Double-blind placebo-controlled randomised phase III trial (*n* = 1366)	Histologically confirmed OC (stage IIB–IV) who had undergone initial debulking surgery	Arm 1: Carboplatin (AUC 5 or 6) plus paclitaxel (175 mg/m^2^) on day 1 every 3 weeks for six cycles combined with either nintedanib 200 mg Arm 2: Placebo BID on days 2–21 every 3 weeks for up to 120 weeks	**mPFS:** Arm 1:17.6 Arm 2: 16.6 (HR: 0.86, 0.75–0.98, *p* = 0.029) **mOS:** Arm 1: 62 Arm 2: 62.8 (HR: 0.99, 0.83–1.17, *p* = 0.86)	PFS improvement seen with nintedanib combination therapy did not affect OS compared with placebo.
TRINOVA-3 [49]	Double-blind study phase III, (*n* = 1015)	Biopsy confirmed OC (stages III-IV)	Arm 1: six cycles of paclitaxel (175 mg/m^2^) and carboplatin (AUC 5 or 6) q3 weeks, plus weekly intravenous trebananib 15 mg/kg Arm 2: 6 cycles of paclitaxel (175 mg/m^2^) and carboplatin (AUC 5 or 6) q3 weeks, plus weekly placebo	**mPFS:** Arm 1: 15·9 Arm 2: 15·0 (HR: 0·93, 0·79–1·09, *p* = 0·36)	Trebananib plus carboplatin and paclitaxel as first-line treatment did not improve PFS in advanced OC.

*AEs* Adverse events, *AUC* Area under the curve, *BID* Twice daily, *CI* Confidence interval, *EOC* Epithelial ovarian cancer, *HR* Hazard ratio, *ITT* Intention-to-treat, *NA* Not applicable, *OC* Ovarian cancer, *OS* Overall survival, *PFI* Platinum-free interval, *mPFS* Median progression-free survival

^a^High-risk of progression was defined as stage IV disease, inoperable stage III disease or sub-optimally debulked (> 1 cm residual) stage III disease

### PARP inhibitors

The approval of PARP inhibitors in 2014 for the management of recurrent EOC resulted in a paradigm shift in the treatment landscape. PARP inhibitors are one of the new class of medications for EOC, targeting the DNA repair fragility of tumor cells. PARP inhibitors have been shown to trap enzymes PARP1 and PARP2 on DNA, leading to PARP-DNA complexes. This “trapping of PARP” potentiates synergism between PARP inhibition and both platinum-based chemotherapy and alkylating agents. However, there are remarkable differences in the PARP inhibitors ability to trap PARP, based on the size and structure of each molecule [50]. Among PARP inhibitors that have already been evaluated, olaparib, niraparib, and rucaparib trap PARP 100-fold more efficiently compared to veliparib, whereas talazoparib appears to be the most potent PARP trapper investigated so far. Increased PARP trapping is found to be associated with high myelosuppression, which possibly results in variation of the recommended doses across PARP inhibitors [51].

The phase III trials, Study-19 [52, 53], SOLO-2 [24, 25], NOVA [26, 27] and ARIEL-3 [23] have demonstrated PFS benefit with PARP inhibitors maintenance therapy (olaparib, niraparib and rucaparib), in platinum-sensitive recurrent OC. Based on the positive results, the US FDA approved PARP inhibitors for the maintenance treatment of recurrent EOC, fallopian tube or primary peritoneal cancer, in patients who are in complete response (CR) or partial response (PR) to platinum-based chemotherapy. The role of PARP inhibitors as maintenance therapy was evaluated in frontline setting in four phase III trials (SOLO-1 [54], PRIMA [55], PAOLA-1 [56] and VELIA [57]). The details of clinical trials with PARP inhibitors maintenance in OC management are summarised in Table 2.

**Table 2:** Clinical trials on PARP inhibitor maintenance treatment in EOC (recurrent and frontline)

Reference	Study design	Study population	Treatment modality and no of patients	Median PFS /OS/ORR in months Hazard Ratio (95% CI)	Conclusion
**In recurrent settings**					
Study-19 [52, 53]	Randomised, double-blind, phase II study	≥2 prior lines of chemotherapy HGSOC platinum-sensitive	Olaparib 400 mg BID capsules (*n* = 136) versus placebo (*n* = 129)	**mPFS:** *▪ BRCA* mutation: 11.2 vs. 4.3 (HR: 0.18, 0.10–0.31) *▪ BRCAwt*: 7.4 vs. 5.5 (HR: 0.54, 0.34–0.85, *p* = 0.0075) *▪* ITT: 8.4 vs. 4.8 (HR: 0.35, 0.25–0.49, *p* < 0.001) **mOS:** *▪* At the second interim analyses, OS did not significantly differ between the groups (HR: 0·88, 0.64–1.21, *p* = 0.44); similar findings were noted for patients with mutated *BRCA*	*▪* In the ITT population PFS benefit was seen irrespective of *BRCA*, Also patients with *BRCA* mutation have the greatest likelihood of benefit from olaparib therapy.
NOVA [26, 27]	Randomised, double-blind, phase III trial	≥2 prior lines of chemotherapy High-grade serous platinum-sensitive HRD testing in *BRCAwt*	Niraparib 300 mg OD (*n* = 372) versus placebo (*n* = 181)	**mPFS:** *▪ BRCA* mutation: 21.0 vs. 5.5 (HR: 0.27, 0.17–0.41) *▪ BRCAwt*, HRD: 12.9 months) (HR: 0.38, 0.24–0.59) *▪ BRCA* cohort (9.3 months) (HR: 0.45, 0.34–0.61, *p* < 0.001 for all three comparisons) **mPFS2:** *BRCA* mutation: HR: 0.67 (0.48 − 0.95) *BRCA*wt: 0.81 (0.62 − 1.05) **mOS:** Restricted mean survival time analyses up to 72 months *BRCA* mutation: 45.9 vs 43.2 (Δ of 2.7 m,: − 4.1 − 9.5) *BRCAwt*: 38.5 vs 39.1 (Δ of − 0.6 m, − 6.0 − 4.7)	*▪* The mPFS duration was significantly longer among those who received niraparib regardless of the presence or absence of *gBRCA* mutations/HRD status, with moderate bone marrow toxicity. *▪* The final data support the safe long-term use of niraparib for maintenance treatment. PFS2 analyses indicates that the benefit of niraparib maintenance therapy extends beyond first progression. The NOVA study was not powered for OS and analyses is confounded by a high rate of crossover and missing data thus limiting its interpretation.
ARIEL-3 [23]	Randomised, double-blind, placebo-controlled, phase III trial	≥2 prior lines of chemotherapy High-grade serous or endometrioid platinum-sensitive	Rucaparib 600 mg BID (*n* = 375) versus placebo (*n* = 189)	**mPFS:** *▪ BRCA* mutation: 16.6 vs. 5.4 (13.4–22.9, *p* < 0·0001) *▪* HRD: 13·6 vs. 5.4 (10.9–16.2, *p* < 0·0001) *▪* ITT population: 10.8 vs. 5.4 (8.3–11.4, *p* < 0·0001)	*▪* Across all primary analyses groups (*BRCA* mutant carcinoma, HRD carcinoma and ITT population), rucaparib substantially improved PFS in patients with platinum-sensitive OC who had achieved a response to platinum-based chemotherapy.
SOLO-2 [24, 25]	Multicentre, double-blind, randomised, placebo-controlled, phase III trial	≥2 prior lines of chemotherapy high-grade serous or endometrioid platinum-sensitive *gBRCA* mutations	Olaparib 300 mg bd tablets (*n* = 196) verses placebo (*n* = 99)	**mPFS:** *BRCA* mutation: Investigator-assessed mPFS: 19.1 vs. 5.5 (HR: 0.30, 0.22–0.41, *p* < 0.001) **mOS:** *▪* A long-term treatment benefit was seen with olaparib vs placebo (51.7 vs. 38.8) with an HR of 0.74 (0.54–1.00, *p* = 0·054)	*▪* Maintenance olaparib provided an unprecedented improvement of 12.9 months in median OS vs placebo in patients with platinum-sensitive, relapsed OC with *BRCA* mutation.
**Frontline settings**					
SOLO-1 [54]	Randomised, double-blind, phase III trial	Stage III/IV HGSOC or endometrioid g BRCA mutations CR or PR to chemotherapy	Olaparib 300 mg BID tablets (*n* = 260), vs. placebo (*n* = 131)	**mPFS:** *▪* After 5 years: 56 vs. 13.8 (HR: 0.33 0.25–0.43)	*▪* The use of maintenance therapy with olaparib provided a substantial benefit with regard to PFS with a 63% lower risk of disease progression or death with olaparib than with placebo. *▪* After 5-years, almost half of patients receiving maintenance olaparib were progression-free compared to with placebo (20%). Over 50% of patients in complete response after first-line platinum-based chemotherapy remained free from relapse 5 years later.
PRIMA [55]	Randomised, double-blind, phase III trial	Stage III (residual disease)/IV HGSOC or endometrioid CR or PR to chemotherapy	Niraparib 300 mg (*n* = 487) vs. placebo (*n* = 246)	**mPFS:** *▪* ITT: 13.8 vs. 8.2 (HR: 0.62, 0.50–0.76, *p* < 0.001) *▪* HRD: 21.9 vs. 10.4 (HR: 0.43, 0.31–0.59, *p* < 0.001) *▪ BRCA* mutation: 22.1 vs. 10.9 (HR: 0.40, 0.27–0.62) *▪ BRCAwt*, HRD: 19.6 vs. 8.2 (HR: 0.50, 0.31–0.83) **mOS:** *▪* At the 24-month interim analyses, the rate of OS in the niraparib group was 84%	*▪* Among patients with newly diagnosed advanced OC who had a response to platinum-based chemotherapy, those who received niraparib had significantly longer PFS, regardless of the presence or absence of HRD.
VELIA [57]	Phase III, placebo-controlled trial	Stage III/IV HGSOC CR, PR or SD to chemotherapy	Carboplatin/taxane plus maintenance placebo (*n* = 375), carboplatin/taxane and maintenance veliparib (*n* = 383) vs. carboplatin/taxane with veliparib and maintenance veliparib (*n* = 382)	**mPFS:** *▪* ITT population: 23.5 vs. 17.3 (HR: 0.68, 0.56–0.83, *p* < 0.001) *▪ BRCA* mutation: 34.7 vs. 22.0 (HR: 0.44, 0.28–0.68, *p* < 0.001) *▪* HRD: 31.9 vs.20.5 (HR: 0.57, 0.43–0.76, *p* < 0.001) *▪ BRCAwt* and HRD: 15.0 vs. 11.5 (HR: 0.74, 0.52–0.76) *▪ BRCAwt*: 18.2 vs. 15.1 (HR: 0.80, 0.64 –1.00)	*▪* Across all study populations, treatment with carboplatin, paclitaxel, and veliparib induction therapy followed by veliparib maintenance therapy led to significantly longer PFS than carboplatin plus paclitaxel induction therapy alone.
PAOLA-1 [56]	Randomised, double-blind, phase III trial	Stage III/IV high-grade serous or non-serous OC with CR or PR to chemotherapy	Olaparib 300 mg (*n* = 537) BID plus bevacizumab (15 mg/kg dL, q3w) versus placebo (*n* = 269) plus bevacizumab	**mPFS:** *▪* ITT population: 22.1 vs. 16.6 (HR: 0.59, 0.49 –0.72, *p* < 0.001) *▪ BRCA* mutation cohort: 37.2 vs 21.7 (HR: 031, 0.20–0.47) *▪* HRD: 37.2 vs. 17.7 (HR:0.33, 0.25–0.45) *▪ BRCAwt* and HRD: 28.1 vs. 16.6 (HR: 0.43, 0.28–0.66) *▪ BRCAwt:* 18.9 vs. 16.0 (HR: 0.71, 0.58–0.88)	*▪* In patients with advanced OC receiving first-line therapy with bevacizumab, the addition of maintenance olaparib provided a substantial PFS benefit, which was significant in patients with HRD-positive tumours, including individuals without a *BRCA* mutation.

*BID* Twice daily, *BRCA BReast CAncer gene*, *BICR* Blinded independent central review, *CI* Confidence interval, *CR* Complete response, *DP* Disease progression, *gBRCA Germline BReast CAncer gene*, *HGSOC* High-grade serous ovarian cancer, *HR* Hazard’s ratio, *HRD* Homologous recombination defect genes, *ITT* Intention-to-treat population, *mPFS* Median progression-free survival, *NACT* Neo-adjuvant chemotherapy, *NC* Not calculated, *OC* Ovarian cancer, *OR* Odds ratio, *ORR* Objective response rate, *OS* Overall survival, *PDS* Primary debulking surgery, *PR* Partial response, *RD* Residual disease, *BRCAwt BRCA wild-type*

In SOLO-1 study [54], patients with newly diagnosed stage III-IV, with positive *BRCA* mutation status showed significant PFS benefit with a 67% risk reduction for disease progression or death in olaparib arm compared with placebo, beyond 5 years (56.0 months vs. 13.8 months, HR: 0.33, 95% CI, 0.25 to 0.43). Olaparib was approved for frontline maintenance therapy in patients with deleterious or suspected deleterious germline or somatic *BRCA*-mutated EOC, fallopian tube or primary peritoneal cancer based on results of SOLO-1 trial [58].

The PRIMA trial [55] investigated the effectiveness of niraparib first-line maintenance therapy in patients with advanced EOC. A significant improvement in PFS was seen with niraparib over placebo, in the overall population (13.8 months vs. 8.2 months, HR: 0.62, 95% CI, 0.50 to 0.76; *p* < 0.001) as well as in homologous recombination deficiency (HRD) cohort (21.9 months vs. 10.4 months, HR: 0.43, 95% CI, 0.31 to 0.59, *p* < 0.001). The homologous recombination-proficient cohort also showed significant improvement in PFS (8.1 months vs. 5.4 months, HR: 0.68, 95% CI, 0.49 to 0.94, *p* = 0.020); however the magnitude of benefit is much lesser than the other groups. The trial confirmed that the clinical benefit with niraparib frontline maintenance therapy could be extended to all patients with advanced EOC, regardless of HRD status. Niraparib is currently approved for the first-line maintenance treatment of patients with advanced EOC, fallopian tube, or primary peritoneal cancer who are in a complete or partial response to first-line platinum-based chemotherapy [59].

The PAOLA-1 study [56] examined the efficacy of combination therapy of PARP inhibitors with bevacizumab as frontline maintenance therapy in patients with advanced EOC, with complete or partial response to standard platinum-based therapy given with bevacizumab. A significant improvement in PFS was demonstrated in the intention-to-treat population with bevacizumab plus olaparib compared to placebo (22.1 months vs. 16.6 months, HR: 0.59, 95% CI, 0.49 to 0.72, *p* < 0.0001). An exploratory analyses, in HRD-positive population, an extended PFS benefit has been observed with olaparib plus bevacizumab compared to placebo plus bevacizumab (37.2 months vs. 17.7 months, HR: 0.33; 95% CI, 0.25 to 0.45); no PFS benefit was witnessed in patients with negative HRD status (16.6 months vs. 16.2 months, HR: 1.00, 95% CI, 0.75 to 1.35). In patients with *BRCA* mutations, an extended PFS has been observed with a 69% risk reduction for disease progression or death in olaparib compared to placebo (37.2 months vs. 21.7 months, HR: 0.31, 95% CI, 0.20 to 0.47) [56]. Olaparib was approved in combination with bevacizumab by the FDA for the first-line maintenance treatment of adult patients with HRD-positive advanced EOC, fallopian tube or primary peritoneal cancer patients who are in CR or PR to first-line platinum-based chemotherapy [58].

The VELIA study [57] assessed the efficacy of veliparib added to first-line therapy with chemotherapy and continued as maintenance monotherapy in patients with newly diagnosed advanced EOC. In the overall population, extended PFS was shown in veliparib cohort (23.5 months vs. 17.3 months, HR: 0.68, 95% CI, 0.56 to 0.83, *p* < 0.001). In patients with *gBRCA* mutation, the median PFS was longer with veliparib (34.7 months vs. 22.0 months, HR: 0.44, 95% CI, 0.28 to 0.68, *p* < 0.001); the benefit was also observed in patients with HRD-positive status (31.9 months vs. 20.5 months, HR: 0.57, 95% CI, 0.43 to 0.76, *p* < 0.001). No benefit was seen in patients with *BRCA* wild-type (*BRCA*wt*)* disease (HR: 0.80, 95% CI, 0.64 to 1.00) or those with homologous recombination-proficient disease (HR: 0.81, 95% CI, 0.60 to 1.09).

All three studies (PRIMA [55], PAOLA-1 [56], VELIA [57]) were affirmative in the overall population; despite specific genetic aberrations, the HRD-positive patients derived most benefit either due to a *BRCA* mutation or other HRD.

#### Novel therapies

In phase III clinical trial (NCT03863860), fuzuloparib (formerly fluzoparib) as maintenance therapy achieved a clinically meaningful and statistically significant improvement in PFS in patients with platinum-sensitive, recurrent OC (12.9 months vs. 5.5 months, 95% CI, 0.17 − 0.36, *p* < 0.0001) compared with placebo. The risk of disease progression or death was reduced by 75% (HR: 0.25) with manageable safety profile regardless of *BRCA* mutation status [60].

##### Immune checkpoint inhibitors

Immune checkpoint inhibitors (ICIs) are drawing attention as drugs that can extend OS. However, the clinical studies on biological maintenance therapies with ICIs have shown neither PFS nor OS benefit [61]. In phase III MIMOSA trial involving stage III-IV OC patients who had complete clinical remission after primary CRS and chemotherapy with platinum and taxane, abagovomab maintenance therapy has showed measurable immune response [62]. However, it did not prolong recurrence-free survival or OS. Several clinical trials for the efficacy of ICIs as first-line maintenance therapy are ongoing (NCT03737643, NCT03038100, NCT03522246) (Table 3). Phase III trials are also currently evaluating combinations of bevacizumab with ICIs in the frontline therapy and maintenance, post to chemotherapy, with data anticipated to emerge over the next 3 years. IMagyn050/GOG 3015/ENGOT OV-39 is one such trial (ICI: atezolizumab, chemotherapy [carboplatin and paclitaxel], which demonstrated no improvement in PFS with ICIs in newly diagnosed OC in the initial results [63].

**Table 3:** Ongoing phase III trials on combination maintenance therapy in frontline settings

Trial	Trial design	ClinicalTrials.gov identifier	No of patients	Patient population	Treatment arms	Primary end point	Estimated study completion date
IMagyn050/GOG 3015/ENGOT-OV39	2-arm, phase III, randomised trial	NCT03038100	1300	Newly diagnosed stage III/IV OC	1. CT + BEV + atezolizumab ➔ BEV + atezolizumab 2. CT + BEV + placebo ➔ BEV + placebo	PFS and OS (ITT and PD-L1+ populations)	December 2022
ATHENA/GOG-3020/ENGOT-OV45	4-arm, phase III, randomised maintenance trial	NCT03522246	1012	Newly diagnosed stage III/IV EOC in response after completing CRS (PDS or IDS) and first-line platinum-based chemotherapy	1. Rucaparib + nivolumab 2. Rucaparib + placebo 3. Placebo + nivolumab 4. Placebo + placebo	Investigator-assessed PFS	December 2024
DUO-O/AGO-OVAR23/ENGOT-OV46	2-cohort, phase III, randomised trial: double-blind 3-arm trial in non-*BRCA*-mutated and single open-label cohort in *BRCA*-mutated	NCT03737643	1374	Newly diagnosed stage III/IV OC, candidate for CRS (PDS or IDS), bevacizumab eligible	1. CT + BEV + placebo ➔ BEV + placebo + placebo 2. CT + BEV + durvalumab ➔ + BEV + durvalumab + placebo 3. CT + BEV + durvalumab ➔ BEV + durvalumab + olaparib 4. Tumour BRCA-mutated cohort: CP + BEV (optional) + durvalumab ➔ BEV (optional) + durvalumab + olaparib	PFS in non-*tBRCA* HRD positive and all non-*tBRCA* patients patients	June 2023
ENGOT-OV43/GOG-3036/KEYLYNK-001	3-arm randomised trial	NCT03740165	1086	Newly diagnosed non-*BRCA*-mutated stage III/IV EOC having undergone PDS or eligible for PDS or IDS, candidate for CP	1. CT + pembrolizumab ➔ pembrolizumab + olaparib 2. CT + pembrolizumab ➔ pembrolizumab + placebo (optional BEV) 3. CT+ placebo ➔ placebo + placebo (optional BEV)	Investigator-assessed PFS and OS	October 2023
FIRST/ENGOT-OV44	3-arm randomised trial	NCT03602859	912	Newly diagnosed stage III/IV non-mucinous EOC	1. CT + placebo ➔ placebo + placebo 2. CT + placebo ➔ niraparib + placebo 3. CT + dostarlimab ➔ niraparib + dostarlimab	Investigator-assessed PFS	July 2023
MAMOC	2-arm randomised trial	NCT04227522	190	Non-*BRCA-*mutated	1. CT + BEV ➔ BEV ➔ rucaparib 2. CT + BEV ➔ BEV ➔ placebo	PFS	January 2023

*BRCA* BReast CAncer gene, *BEV* Bevacizumab, *CT* Chemotherapy, *CRS* Cytoreductive surgery, *EOC* Epithelial ovarian cancer, *IDS* Interval debulking surgery, *HRD* Homologous recombination deficiency, *ITT* Intention-to-treat population, *OS* Overall survival, *OC* Ovarian cancer, *PDS* Primary debulking surgery, *PFS* Progression-free survival, *PDL-1* Programmed death-ligand 1, *tBRCA* Tumour BReast CAncer gene

## Considerations for maintenance therapy

### Tumour histology

Although EOC is treated as a single entity, each subtype is associated with a discrete clinical behaviour including pattern of metastases, response to systemic chemotherapy and survival [64]. The histological grading (0-3) created based on response to neo-adjuvant chemotherapy in the basis of degree of disappearance of cancer cells, displacement by necrotic and fibrotic tissue and tumour-induced inflammation showed significant association of histological grades 0-1 (HR: 1.65, *p* = 0.03) with reduced OS. The analyses also confirmed histological grades 0-1 (odds ratio [OR]: 8.42, *p* = 0.003) as independent predictors of relapse within 6 months [65]. In serous ovarian tumours, the high-grade tumours are found to be associated with shorter OS than low-grade serous cancers [66]. High-grade serous ovarian carcinoma (HGSOC) is known to be associated with higher incidence of *BRCA* mutations [67]; they have the best response to PARP inhibitors [56]. Interpretation of cellular morphology defines the EOC subtypes and guides appropriate treatment planning based on tumour and patient characteristics, moreover it may also help in understanding the potential need for maintenance therapy [68].

### Molecular status and testing

HRD, a lack of functional components in one or more of the DNA repair pathways like the HRR, is a common feature of OC, especially in HGSOC. *BRCA* mutations (germline or somatic) are the most prevalent mutations among *HRR* genes (germline or somatic mutations). Testing for *BRCA* mutation has proved to be an effective diagnostic and prognostic tool in OC [69], as demonstrated by the efficacy of platinum-based drugs in this disease and the advent of PARP inhibitors for the maintenance treatment of these patients with mutations in *HRR* genes [19, 20, 70]. In a systematic review of 33 studies in patients with primary or recurrent OC (*n* = 7745) significantly longer PFS (HR: 0.80; 95% CI, 0.64 to 0.99, *p* = 0.039) and OS (HR: 0.75, 95% CI, 0.64 to 0.88, *p* < 0.001) were reported in *BRCA1/2* mutation carriers in response to platinum-based chemotherapy [20]. In patients with OC, *BRCA*-mutated patients had a significant PFS benefit compared with *BRCA*wt cancer (HR: 0.65; 95% CI, 0.44 to 0.98, *p* = 0.032) with PARP inhibitors; with no significant difference in somatic and germline mutations carriers [19]. Similar results were reported in another study in patients with HGSOC [70]. This effect has also been observed in patient with HRD [71]. Patients with HRD have a better response possibly because of the synergism of cell-damaging effects. In newly diagnosed advanced OC, higher HRD scores have been associated with improved PFS, indicating a prognostic significance to this marker [72]. It is thus imperative to provide genetic testing for HRD and *BRCA* for making treatment decisions regarding evaluation of response to chemotherapy or targeted therapy or PARP inhibitor maintenance therapy. Medical societies recommend *BRCA* testing for all patients diagnosed with OC [22]; however, HRD testing may not lag behind because the clinical validity is assessed in terms of PARP inhibitor benefit rather than in terms of biological HRD status. Hence, HRD status is not routinely tested in many countries. Recently, it was reported that *HRR* mutation analyses should not be considered as a substitute for HRD determination by *BRCA* or genomic instability testing, since *HRR* mutation gene panels failed to demonstrate its utility beyond tumour *BRCA* mutation for selecting patients who may benefit from maintenance olaparib plus bevacizumab in PAOLA-1 trial [73].

The next generation sequencing (NGS) panel, consisting of multiple genes, can detect different genetic aberrations, point mutations, indels and copy number variations in a single test, in short turnaround times. *BRCA* tumour testing by NGS simultaneously detect both somatic and germline mutations, allowing the identification of more patients with higher likelihood of benefiting from PARP inhibitors. The NGS gene panels are customisable and provide flexibility to select the therapeutically actionable genes. Companion diagnostics can play an important role in selecting the genes for NGS testing. MyChoice^®^ CDx (Myriad^®^ Genetics Inc) was used in PAOLA- 1[56], PRIMA [55] and VELIA [57] trials to select patients who were most likely to derive therapeutic benefit from these PARP inhibitors. For rucaparib FoundationFocus™ CDx_*BRCA LOH*_ (Foundation Medicine) was utilised to detect somatic *BRCA* mutations [23]. Maintenance treatment with targeted agents in advanced OC can be cost-effective, when guided by companion diagnostics.

### Safety considerations

Though the clinical benefit of maintenance therapy with anti-angiogenic and PARP inhibitors in the frontline setting is evident, they do carry a risk for toxicity resulting in dose interruptions and reductions. The adverse events (AEs) associated with bevacizumab treatment are hypertension, proteinuria, headache and epistaxis and less commonly taste alteration, rhinitis, dry skin, rectal haemorrhage, exfoliative dermatitis, and lacrimation disorder [74]. The most common > = 3 AEs that occurred at a higher incidence in phase III randomised trials for niraparib, olaparib and veliparib were anaemia followed by thrombocytopenia, neutropenia and fatigue/asthenia (Table 4) [28, 55–57]. The incidence of grade > =3 AEs was notably higher in the experimental arm compared with placebo arm in PRIMA [55] and VELIA [57] trials and to a lesser extent in the SOLO-1 trial [28]. In PRIMA [55] and VELIA [57] trials, this elevated incidence was driven by frequent grade > =3 haematological AEs and haematological toxicities, whereas in SOLO-1 [28], the most common grade > =3 AE was anaemia. In the PAOLA-1 [56], incidences of grade > =3 AEs went beyond 50% in both olaparib plus bevacizumab and placebo plus bevacizumab maintenance regimen. However, addition of olaparib to bevacizumab did not increase bevacizumab-associated toxicity. Hypertension was the most frequent grade > =3 AE in PAOLA-1 and olaparib did not seem to increase this classic bevacizumab-associated toxicity; in fact, the olaparib-containing arm was associated with lower incidences of all-grade and grade > =3 hypertension compared with the bevacizumab-alone arm [56]. The patients receiving a combination with chemotherapeutic regimen were found to be at a higher risk of haematologic toxicities [75]. Risk of treatment-induced acute myeloid leukaemia and myelodysplastic syndrome for olaparib and niraparib was reported to be < 1.5 and 0.9% respectively [58, 59]. Fatigue, gastrointestinal problems and haematologic toxicities are the common low-grade AEs reported for PARP inhibitors treatment in patients with EOC. The proportion of patients with AEs leading to treatment discontinuation was high with olaparib plus bevacizumab maintenance (20%) compared with niraparib (12%), olaparib (12%) and veliparib maintenance (19%), whereas dose reduction was high (70%) with niraparib [28, 55–57]. In health-related quality of life, no clinically significant change has been observed between the PARP inhibitors maintenance and placebo in PRIMA [55], SOLO-1 [28], PAOLA-1 [56] and VELIA [57] trials. Initiation of prophylactic supportive treatments and dose interruptions may allow resumption of the drugs at the same suggested dose level. The AE profile and the clinical status of the patient should be considered while selecting and initiating therapy with PARP inhibitors [76].

**Table 4:** Summary of safety in phase III trials of PARP inhibitors maintenance therapy in frontline settings

Trial	PRIMA [55] (*n* = 728)		SOLO-1 [28] (*n* = 390)		PAOLA-1 [56] (*N* = 802)		VELIA [57] (*N* = 621)	
	Niraparib (*n* = 484)	Placebo (*n* = 244)	Olaparib (*n* = 260)	Placebo (*n* = 130)	Olaparib + Bevacizumab (*n* = 535)	Placebo (*n* = 267)	Veliparib^a^ (*n* = 310)	Placebo (*n* = 311)
Any grade, n (%)	478 (99)	224 (92)	256 (98)	120 (92)	531 (99)	256 (96)	294 (95)	290 (93)
Grade > =3^b^, n (%)	341 (70)	46 (19)	102 (39)	24 (18)	303 (57)	136 (51)	138 (45)	99 (32)
AE leading to treatment discontinuation, n (%)	58 (12)	6 (2)	30 (12)	3 (2)	109 (20)	15 (6)	58 (19)	3 (1)
AE leading to dose reduction, n (%)	343 (71)	20 (8)	74 (28)	4 (3)	220 (41)	20 (7)	74 (24)	12 (4)
Selected grade > =3, n (%)								
Anaemia	150 (31)	4 (2)	56 (22)^c^	2 (2)^c^	93 (17)^c^	1 (< 1)^c^	23 (7)	3 (1)
Thrombocytopenia	139 (29)	1 (< 1)	2 (1)^d^	2 (2)^d^	9 (2)^d^	1 (< 1)^d^	20 (6)	1 (< 1)
Neutropenia	62 (13)	3 (1)	22 (8)^e^	6 (5)^e^	32 (6)^e^	8 (3)^e^	16 (5)	12 (4)
Fatigue/asthenia	9 (2)	1 (< 1)	10 (4)	2 (2)	28 (5)	4 (1)	19 (6)	3 (1)

*AE* Adverse event, *PARP* Poly (ADP-ribose) polymerase inhibitor

^a^Data are reported only for the veliparib-throughout and control arms, excluding the veliparib combination-only arm

^b^Excludes grade 5 in SOLO-1 and VELIA

^c^Includes anaemia, decreased haemoglobin level, decreased haematocrit, decreased red cell count, erythropenia, macrocytic anaemia, normochromic anaemia, normochromic normocytic anaemia and normocytic anaemia

^d^Includes thrombocytopenia, decreased platelet production, decreased platelet count and decreased plateletcrit

^e^Includes neutropenia, febrile neutropenia, neutropenic sepsis, neutropenic infection, decreased neutrophil count, idiopathic neutropenia, granulocytopenia, decreased granulocyte count and agranulocytosis

According to real-world evidence from the US healthcare claims data focusing on comparative tolerability and dose modifications in patients with OC receiving PARP inhibitor therapy, the risk of experiencing any clinical events of interest (CEI) was significantly higher with niraparib compared with olaparib (OR: 3.23, 95% CI, 1.89 to 5.50, *p* < 0.001) and rucaparib (OR: 2.07, 95% CI, 1.08 to 3.97, *p* < 0.05), with no significant difference between rucaparib and olaparib (OR: 1.56, 95% CI, 0.89 to 2.74, *p* = 0.1). A similar pattern was reported with haematologic CEIs. PARP inhibitor dose decreases were observed in 21.1, 30.2 and 35.1% of olaparib-, rucaparib- and niraparib- treated patients, respectively [77]. In a comparative study evaluating efficacy and tolerability of olaparib, niraparib and rucaparib in *BRCA*-mutated platinum-sensitive relapsed OC, olaparib demonstrated superior tolerability with reduced odds for grade 3-4 AEs compared with niraparib and rucaparib and a superior tolerability than niraparib for dose reduction [78].

### Frontline versus recurrent maintenance – the quintessential paradox

**Fig. 1:**
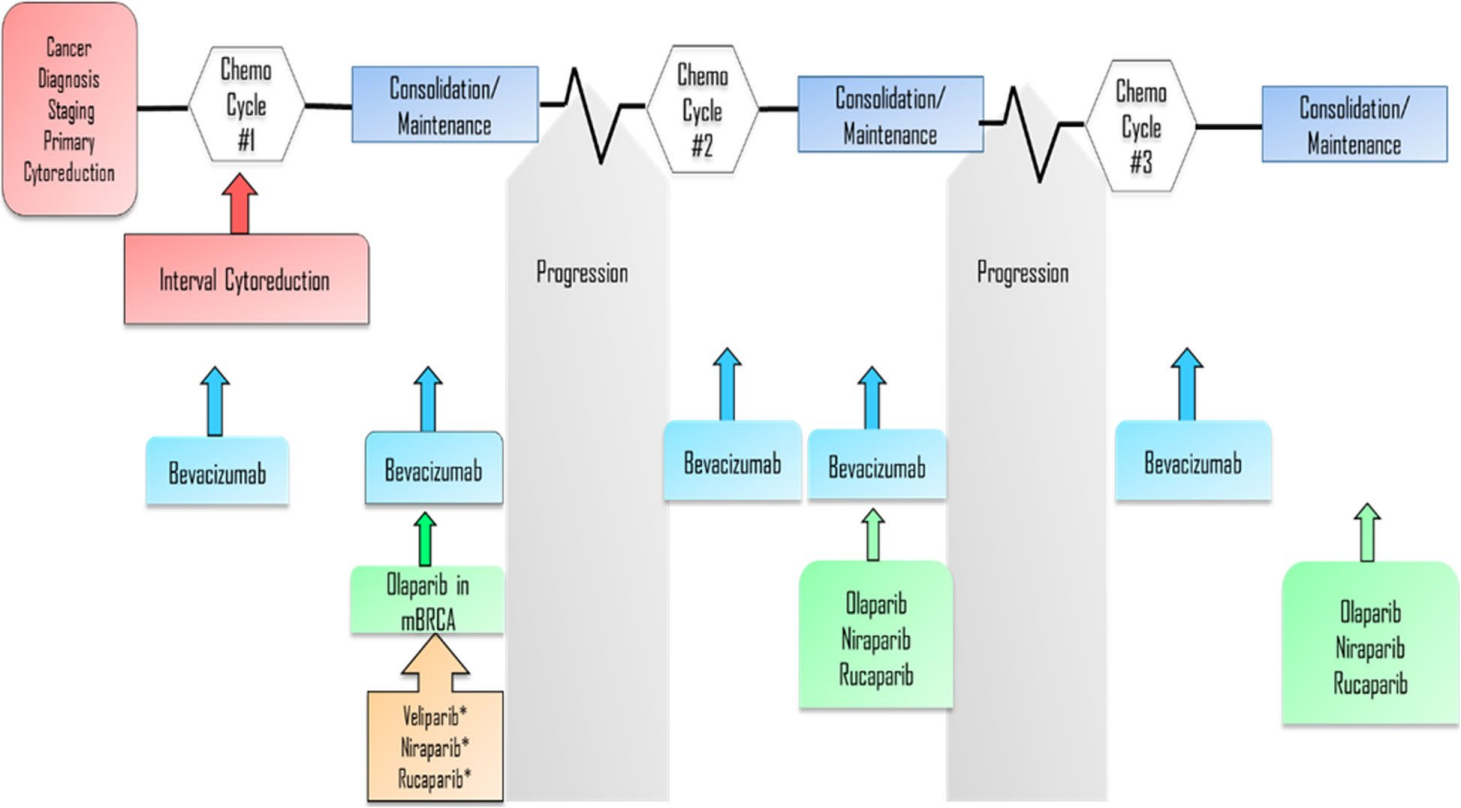
Evolution of Maintenance Strategy for Epithelial Ovarian Cancer *Future prospects

The current treatment landscape for OC has transformed greatly compared with the past decade with the advent and approval of novel therapies. Figure 1 illustrates the evolution of treatment strategies in the management of EOC. The choice of maintenance therapy in frontline or recurrent settings in patients with advanced OC vary based on patient’s clinical features, molecular status, initial therapy and patient’s preferences. Treatment discontinuation is frequently observed with increased lines of therapy in patients with advanced OC [79]. A real-world study reported that approximately half of the treated cohort having a treatment discontinuation or death within the first 4 month or transfer to second-line or later therapies within a few months of initiation of the first-line therapy [80]. A majority (75%) of the patients received standard chemotherapy for advanced disease [80].

Also with multiple relapses, PFS time shortens following each recurrence and subsequent round of therapy (after the first, second, third, fourth and fifth relapse PFS was 10.2, 6.4, 5.6, 4.4 and 4.1 months, respectively) [81]. In advanced cancer, patients may respond well to first-line therapy, but then progress and deteriorate so rapidly that they are unable to receive second-line therapy. Hence, maintenance therapy after induction therapy in frontline setting could be very beneficial in improving survival rates. SOLO-2 trial conducted on patients with platinum-sensitive, relapsed OC and a *BRCA* mutation has confirmed an OS benefit with olaparib maintenance therapy [24]. Although the improvement in OS with olaparib maintenance therapy was not statistically significant, it was clinically meaningful [24]. The observed PFS benefit in newly diagnosed OC could be possibly due to the introduction of PARP inhibitors at first-line therapy [54]. This could limit the number of patients likely to expire at first tumour progression, along with platinum-resistant relapse within 6 months after the end of chemotherapy and those who would not be benefited from PARP inhibitors during recurrence. With this intent, the NCCN Clinical Practice Guidelines in Oncology (NCCN Guidelines^®^) recommend PARP inhibitors as frontline maintenance therapy options in certain patients with EOC regardless of *BRCA* status [22]. For patients who did not receive bevacizumab during primary therapy and had CR or PR, the NCCN recommends niraparib therapy as an option in patients with *BRCA*wt or an unknown status, and olaparib or niraparib as treatment option in patients with *BRCA1/2* mutations. In patients with *BRCA*wt or an unknown status, who had CR or PR and received bevacizumab as a part of primary therapy, bevacizumab alone is recommended as an option for HR proficient or status unknown, and a combination of bevacizumab and olaparib maintenance therapy is recommended as an option for those with HR deficiency. Whereas for patients with *BRCA1/2* mutations in CR or PR who received bevacizumab as part of primary therapy, a combination of bevacizumab and olaparib maintenance therapy or olaparib/niraparib alone maintenance therapy are recommended as options [22].

### PARP inhibitor combination therapy

In addition to its role as a monotherapy, PARP inhibitor have also proved its use in combination with other DNA-damaging agents, such as chemotherapy and radiation therapy by preventing repair of treatment-induced DNA damage [82]. With the approval of bevacizumab in combination with olaparib, combination therapies with PARP inhibitors are being actively studied. A combination of PARP inhibitors with angiogenesis inhibitors in OC has been studied in several clinical trials [83–86]. The PARP inhibitor–ICI combination has gained more attention with increased programmed cell death protein-1/programmed death-ligand 1 expression, and lymphocyte infiltration in *gBRCA* mutated HGSOC compared with *BRCA*wt disease. The MEDIOLA trial (phase I/II) demonstrated a 70% response rate with olaparib and durvalumab combination therapy in patients with relapsed, platinum-sensitive, *BRCA*-mutated OC [87].

In the OVARIO study (phase II trial), the addition of niraparib maintenance to first-line platinum-based chemotherapy with bevacizumab demonstrated clinical benefit in patients with advanced OC [88]. In the frontline setting, five ongoing clinical trials (KEYLYNK-001/ENGOT-OV43/MK-7339-001 [Pembrolizumab, Olaparib], FIRST/ENGOT-OV44 [niraparib plus TSR-042], ATHENA [(rucaparib and nivolumab], DUO-O [durvalumab-olaparib], ENGOT-OV39 [atezolizumab, bevacizumab] are investigating a combination of PARP inhibitors and ICIs as first-line maintenance therapy after platinum-based chemotherapy (Table 3).

Apart from the combinations of PARP inhibitors with angiogenesis inhibitors and immune checkpoint inhibitors, other inhibitors that specifically inhibit homologous recombination, such as PI3K-, AKT-, mTOR-, WEE1-, MEK-, and CDK4/6 inhibitors may also be effectively combined with PARP inhibitors [89]. Therapeutically, to sensitize OC with HR proficiency (de novo or acquired) to PARP inhibitors, combinations of PARP inhibitors with drugs that inhibit HR might be an effective approach. In the clinical practice, the target is to reduce overlapping toxicities by optimizing the dose and treatment schedule and use combinations in selected patients who would not benefit from PARP inhibitor monotherapy [89]. Beside, several other novel therapies currently being studied for management of EOC include autologous tumour vaccine (Vigil) [90, 91] and dendritic cell vaccine (SOTIO^®^ DCVAC) [92].

### Cost implications

Generally, individuals with cancer need to pay a greater percentage of their treatment costs through coinsurance and deductibles [93]. In most of the cancer patients out-of-pocket cost is a main barrier in starting and adhering to suggested advanced treatments [94]. PARP inhibitors are expensive compared with other available therapies. The out-of-pocket charges may differ depending on the insurance coverage of the patient and the local reimbursement policies. Although most insurance companies arrange for some coverage for PARP inhibitors, the patient’s co-payment may remain unaffordable. The cost of coverage and the size of co-payment may vary geographically. The cost-effective analyses study conducted by Gonzalez et al., reported that universal PARP inhibitor maintenance treatment is cost-effective compared with a biomarker-directed PARP inhibitor strategy [95]. The economic analyses conducted by Tan et al., demonstrated that olaparib has a high potential (87% probability) of being a cost-effective maintenance treatment in Singapore than routine surveillance among patients with advanced OC with *BRCA* mutations after response to first-line chemotherapy at a willingness-to-pay of Singapore dollar 60,000 per quality-adjusted life-year gained [96].

## Conclusion

Antiangiogenic agents and PARP inhibitors have the potential to bridge the unmet need in the management of EOC. Bevacizumab as maintenance treatment has proven its benefit in patients with newly diagnosed advanced EOC at high-risk of disease progression. The use of PARP inhibitors as maintenance with olaparib or niraparib after first-line chemotherapy has shown a significant PFS benefit in the *BRCA* mutations. The combination maintenance treatment with bevacizumab and PARP inhibitor, olaparib, following first-line chemotherapy has demonstrated encouraging improvement in PFS in the *BRCA*-mutated and also in the HRD population. Genetic profiling is providing the necessary insights required to determine the sequencing of the available therapies for patients with EOC and help derive maximum benefit. Identification of biomarkers that predict resistance and combination therapies that can help overcome it may prove beneficial. Thus, in the era of personalised cancer medicine, PARP inhibitor maintenance therapy promises to optimise the management and improve outcomes for patients with EOC.

## Data Availability

Not applicable.

## Abbreviations

AE: Adverse event
*BRCA*: *BReast CAncer gene*
*BRCA*wt: *BRCA* wild-type
CEI: Clinical events of interest
CI: Confidence interval
CR: Complete response
CRS: Cytoreductive surgery
EOC: Epithelial ovarian cancer
FDA: Food and Drug Administration
*gBRCA*: Germline *BRCA*
HGSOC: High-grade serous ovarian carcinoma
HR: Hazard ratio
HRD: Homologous recombination deficiency
HRR: Homologous recombination repair
ICI: Immune checkpoint inhibitor
NCCN^®^: National Comprehensive Cancer Network^®^
NGS: Next generation sequencing
OC: Ovarian cancer
OR: Odds ratio
OS: Overall survival
PARP: Polyadenosine diphosphate -ribose polymerase
PFS: Progression-free survival
PR: Partial response
US: United States
VEGF: Vascular endothelial growth factor
